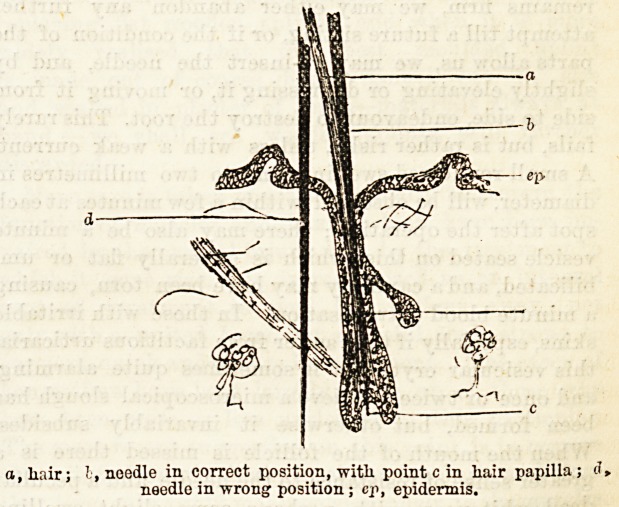# The Electrolytic Treatment of Hirsuties and of Trichiasis

**Published:** 1894-02-17

**Authors:** William Calwell

**Affiliations:** Physician Ulster Hospital for Women and Children, and to Throat Department, Eye, Ear, and Throat Hospital, &c.


### MEDICAL PROGRESS AND HOSPITAL CLINICS.

[*The Editor will be glad to receive offers of co-operation and contributions from members of the profession. All letters should be addressed to* THE EDITOR, THE LODGE, PORCHESTER SQUARE, LONDON, W.]

### THE ELECTROLYTIC TREATMENT OF HIRSUTIES AND OF TRICHIASIS.

By WILLIAM CALWELL, M.A., M.D., Physician Ulster Hospital for Women and Children, and to Throat Department, Eye, Ear, and Throat Hospital, &c.

The removal of superfluous hairs by electrolysis is now recognised as the only permanent method. There are various forms of hirsuties, but some only require treatment; and the disfigurements that are most frequently brought under the notice of a medical man are those situated on the face. Occasionally we are called upon to treat them on the shoulders, arms, and neck; but moustaches and beards on women, and dark moles on the face with long growing hairs, form more than 90 per cent. of the cases about which we are consulted. Shaving, epilation, depilatories answer in an emergency, their repeated practice however entails a still more exuberant growth. The difficulty that fronts us is the same that we meet in ringworm; we must act on the hidden portion of hair embedded in the skin. The following simple experiment will serve to show how electrolysis accomplishes our object. The electrodes of an ordinary constant current battery placed in salt and water cause an evolution in minute bubbles of hydrogen gas at the negative pole, and of oxygen at the positive pole; the salts are likewise decomposed, the bases appearing at the negative pole, the acids at the positive. If the terminations of our electrodes are ordinary steel needles, we notice that the positive needle becomes blackened from the oxidation of iron, and find that this discoloration comes off on our fingers; on the other hand, the negative needle remains bright. When the needles are placed in any animal product, such as white of egg, the same phenomena are presented. The albumen is destroyed, and a large white viscid, frothy clot forms at the negative pole, a smaller discoloured and somewhat adherent clot at the positive. Blood, the tissues of a fibroid tumour, any animal tissue, in fact, are affected in the same way; there is actual destruction in immediate proximity to the needle. If we insert a negative needle down a hair follicle till its point pierces the papilla, and complete the current by the application of a broad positive electrode to the skin, the same tissue destruction takes place in the soft hair root as we have seen in the white of egg.

The practical working of the method is comparatively easy, but there are many details to be carefully attended to in carrying it out to a successful issue. Neglect of these might lead to somewhat unpleasant results, and although some may appear trifling, practice will soon show that they cannot be disregarded. An ordinary constant current battery composed of about nine small Leclanché cells will suffice; not more than four or six are generally used. A galvanometer to measure the strength of the current is an advantage, but not indispensable. Affixed to the positive pole there is a common hand electrode, the negative pole is terminated by a needle-holder with a No. 16 needle, the patient is placed facing a good light, and the part to be operated on steadied in an easy position. The wooden handle of the positive electrode is to be held by the patient in one hand and applied firmly either to the palm of the other hand, or to the cheek if we are operating on the face, or to any other suitable place when word is given, and to be removed also by signal. It is less painful first to insert the needle, and then to get the patient to make and break the current by the application and removal of the positive pole, than to apply the positive pole and then insert or withdraw the needle; besides, we can introduce the needle with much greater exactitude if the disturbance caused by the current is not yet present. The positive electrode must be applied firmly to the skin; if applied too lightly an effect is produced of the current being frequently made and broken, which is painful, and apt to give rise to muscular contractions or the "jumps," as one patient expressed it.

*a*, hair; *b*, needle in correct position, with point *c* in hair papilla; *d*, needle in wrong position; *ep*, epidermis.

If the length of the hair interferes it may be cut short, about one-sixth of an inch. The accompanying diagram illustrates in outline the hair follicle and the correct position of the needle. We must know its depth, which we can judge from the length of root of any adjacent hair, and must be mindful that our needle is parallel to the hair as it enters the skin so that it will likewise lie in the follicle. Having the needle in proper position we give the signal "to touch." Patients sometimes complain of the brassy taste that is experienced when the electrodes are on opposite sides of the chin, also of increased salivation, of subsequent neuralgia in the teeth, and, if the current be rather strong, of flashes of light; but these troubles are seldom of any consequence. It is well to emphasise affixing the needle to the negative pole; if we use the positive pole, a small dark spot will remain after each insertion. On completing the circuit, we notice minute bubbles rising around the needle, and in from five to fifteen seconds, according to the strength of the current, the needle is withdrawn, and the hair should allow of removal with perfect ease; no traction should be requisite. It is better to have a fairly strong current requiring a short time, than to have a weak one necessitating thirty or forty seconds, but we must remember that the former is the sharper weapon, and capable of doing more harm, as it is of doing more good. If the hair is small, however, a weak current is sufficient, and in this case we must be particular that we do not insert the needle too deeply; slight puckering, due to the avoidable tissue destruction, ensues from this fault. A dexterous manipulator may catch the hair with an epilation forceps with one hand, while holding the needle in position with the other, and by employing the very gentlest traction may remove it at the earliest, so that this tissue destruction will be a minimum. In the ordinary way we may allow the electrolysis to proceed after the hair bulb has been destroyed, and in cases of a large number of closely set hairs on a fine skin, every effort must be made to involve the least possible unnecessary tissue destruction, as this will result in scarring. But it is better, perhaps, not to have recourse to this expedient without some little practice, as it may disturb the position of the needle, which is of all importance. In case the hair remains firm, we may either abandon any further attempt till a future sitting, or if the condition of the parts allow us, we may re-insert the needle, and by slightly elevating or depressing it, or moving it from side to side, endeavour to destroy the root. This rarely fails, but is rather risky, unless with a weak current. A small round red swelling, one to two millimetres in diameter, will be observed within a few minutes at each spot after the operation; there may also be a minute vesicle seated on this, which is generally flat or umbilicated, and a capillary may have been torn, causing a minute blood extravasation. In those with irritable skins, especially if they suffer from factitious urticaria, this vesicular erythema is sometimes quite alarming, and once or twice I believe a microscopical slough has been formed, but otherwise it invariably subsides. When the mouth of the follicle is missed there is a greater sense of resistance to the needle, and a peculiar dead whiteness, with perhaps some slight swelling immediately appears; this spreads centrifugally, and is due to the bubbles lifting the horny layer; the needle may be held fast, and some little force required for its withdrawal; this, however, should be done at once, and no further attempt made till next sitting. A lens such as is used in the focal illumination of the eye or an ordinary laryngeal mirror may be employed with advantage, but if the operator is not well accustomed to their use they may only impede him. Warm douching of the part is very soothing, and helps to allay the irritation and inflammation. The swelling may continue for upwards of a week, and where many sittings are indispensable, it is well not to have them more than fortnightly, with occasional intervals of a couple of months, which are also of use in allowing a patient to regain her nerve. The number of hairs removed at each sitting is variable; 10 to 40 may be considered the limits, and a sitting should not last longer than an hour, but this length may cause sleeplessness for a night or so in some.

Those hairs that grow again are generally twisted; if, however, we are successful, no trace of them should be seen, and, unless the hairs are in large numbers and close together, no visible mark is left; when this condition is present, we may make up our minds to some faint scarring. Owing to the danger pointed out above, two hairs close together, about two or three millimetres apart, should never be removed at the same sitting, as it increases the danger of a slough and a linear cicatrix. We may have no visible depression, but only a peculiar white speckling, and this will be more marked in those of dark complexion.

In addition to the bathing with warm water, ladies should wear a veil when exposed to the sun or east wind, and should employ some white sedative astringent lotion, such as the following: ℞ bismuthi carbonat., ʒii.; glycerin., ʒiii.; eau de Cologne, ʒiii.; Aq. ad. ℥vi., ɱ., Ft. lot. (Sig.) To be applied twice daily to part after shaking the bottle. Any white scarring may be relieved by some calamine lotion.

It is hard to foretell how far we should go in cases of a large number of hairs, such as beards or moustaches. Brocq's advice is to let the latter alone. But we may approximately count upon two hundred from the chin with scarcely perceptible marking unless on close inspection. The soft downy hairs which are exceedingly fine and lightly pigmented should not be attempted; it is a great strain on the operator's eyes to detect the follicle, and successful extraction is the exception. Lastly, a little gentle massage for a couple of months or so tends to lessen any stiffness or scar appearance, where a very large number of thickly growing hairs have been removed, but it must be remembered that this will tend to promote the growth of intact roots.

In operating on in-growing eyelashes the same procedure is followed. We can evert either lid sufficiently for our purpose by drawing the palpebral skin either upwards or downwards with the thumb, and at the same time we can thus steady it. The chief troubles are the spasm and the flow of tears, and we can only overcome the former by pretty firm pressure on the margin of the orbit, and the latter by frequent drying. Deep, sunken eyes require considerable patience. It is not advisable to attempt the treatment of trichiasis when all the eyelashes of the lid are affected. In such cases some of the ordinary remedial operations of ophthalmic surgery should be first undertaken, and then, if there are still eyelashes that give trouble, they can be most advantageously removed by electrolysis. In one case repeated operations had been performed on all four eyelids with very satisfactory results, with the exception of about a couple of dozen hairs. These yielded most readily in a few sittings, and, in cases where the in-growing hairs are the exception, this method is undoubtedly the correct one.

It is sometimes said that it is impossible to remove the minute, faintly-coloured lashes that are so troublesome in chronic cases of entropion, but this is decidedly a mistake; a good light, a magnifying lens, and perhaps a second or third attempt will weed them out. The current should be strong, and the electrolytic action allowed to proceed for double or treble the ordinary length of time, as we cannot possibly hope that the needle will lie in the follicle.

## Figures and Tables

**Figure f1:**